# From Better Diagnostics to Earlier Treatment: The Rapidly Evolving Alzheimer’s Disease Landscape

**DOI:** 10.3390/medicina61081462

**Published:** 2025-08-14

**Authors:** Anastasia Bougea, Manuel Debasa-Mouce, Shelly Gulkarov, Mónica Castro-Mosquera, Allison B. Reiss, Alberto Ouro

**Affiliations:** 1 Medical School, National and Kapodistrian University of Athens, 10679 Athens, Greece; abougea@med.uoa.gr; 2 NeuroAging Group (NEURAL), Clinical Neurosciences Research Laboratory (LINC), Health Research Institute of Santiago de Compostela (IDIS), 15706 Santiago de Compostela, Spain; manuel.debasa@rai.usc.es (M.D.-M.); monicacastro.mosquera@usc.es (M.C.-M.); 3 Department of Foundations of Medicine, NYU Grossman Long Island School of Medicine, Mineola, NY 11501, USA; shellygulk1234@gmail.com; 4 Department of Medicine, NYU Grossman Long Island School of Medicine, Mineola, NY 11501, USA; 5 Centro de Investigación Biomédica en Red en Enfermedades Neurodegenerativas (CIBERNED), Instituto de Salud Carlos III, 28029 Madrid, Spain

**Keywords:** Alzheimer’s disease (AD), biomarkers, amyloid hypothesis, BBB, APOE

## Abstract

*Background and Objectives*: Over the past few years, there has been a significant shift in focus from developing better diagnostic tools to detecting Alzheimer’s disease (AD) earlier and initiating treatment interventions. This review will explore four main objectives: (a) the role of biomarkers in enhancing the diagnostic accuracy of AD, highlighting the major strides that have been made in recent years; (b) the role of neuropsychological testing in identifying biomarkers of AD, including the relationship between cognitive performance and neuroimaging biomarkers; (c) the amyloid hypothesis and possible molecular mechanisms of AD; and (d) the innovative AD therapeutics and the challenges and limitations of AD research. *Materials and Methods*: We have searched PubMed and Scopus databases for peer-reviewed research articles published in English (preclinical and clinical studies as well as relevant reviews and meta-analyses) investigating the molecular mechanisms, biomarkers, and treatments of AD. *Results*: Genome-wide association studies (GWASs) discovered 37 loci associated with AD risk. Core 1 biomarkers (α-amyloid Aβ42, phosphorylated tau, and amyloid PET) detect early AD phases, identifying both symptomatic and asymptomatic individuals, while core 2 biomarkers inform the short-term progression risk in individuals without symptoms. The recurrent failures of Aβ-targeted clinical studies undermine the amyloid cascade hypothesis and the objectives of AD medication development. The molecular mechanisms of AD include the accumulation of amyloid plaques and tau protein, vascular dysfunction, neuroinflammation, oxidative stress, and lipid metabolism dysregulation. Significant advancements in drug delivery technologies, such as focused Low-Ultrasound Stem, T cells, exosomes, nanoparticles, transferin, nicotinic and acetylcholine receptors, and glutathione transporters, are aimed at overcoming the BBB to enhance treatment efficacy for AD. Aducanumab and Lecanemab are IgG1 monoclonal antibodies that retard the progression of AD. BACE inhibitors have been explored as a therapeutic strategy for AD. Gene therapies targeting APOE using the CRISPR/Cas9 genome-editing system are another therapeutic avenue. *Conclusions*: Classic neurodegenerative biomarkers have emerged as powerful tools for enhancing the diagnostic accuracy of AD. Despite the supporting evidence, the amyloid hypothesis has several unresolved issues. Novel monoclonal antibodies may halt the AD course. Advances in delivery systems across the BBB are promising for the efficacy of AD treatments.

## 1. Introduction

Alzheimer’s disease (AD) is the predominant type of dementia that affects individuals older than 65 years old, leading to memory loss, executive impairment, and, ultimately, the inability to perform everyday tasks [1]. Patients with mild cognitive impairment (MCI) show an amyloid pathology in 51% of cases and a non-AD pathology in 30%, with only 19% lacking a neurodegenerative pathology [2]. Clinical diagnostic accuracy declines in atypical presentations, early disease stages, the community, and comorbidities [3]. Up to 39% of patients diagnosed with AD are later confirmed to exhibit an AD pathology, while 30% have a non-AD pathology [4]. Additionally, dementia imposes a significant economic burden, with an estimated annual cost of USD 1 trillion worldwide—a figure expected to rise due to the exponential increase in cases, posing a substantial challenge to healthcare systems [5].

The probabilistic nature of an in vivo AD diagnosis and its postmortem verification make it difficult to detect AD in the early stages when treatment options are most effective. However, recent advances in biomarker research have revolutionized the field of AD diagnosis, providing clinicians with powerful tools to accurately identify the disease at its earliest stages.

Biomarkers in AD are measurable indicators of biological processes in the body, such as gene expression, protein levels, or biochemical pathways, even before symptoms become apparent. A “research framework,” often referred to as the AT(N) framework, was developed in 2011 by the National Institute on Aging and the Alzheimer’s Association for the preclinical, mild cognitive impairment (MCI), and dementia stages of AD [6,7]. The AT(N) research framework concentrated on biomarkers in living individuals, which were categorized into those that indicate β amyloid (Aβ), or “A,” pathologic tau, or “T,” and neurodegeneration, or “(N)”. These biomarkers can be evaluated in vivo and are defined by the underlying pathologic processes in the pathology of AD that characterize the disease at postmortem examinations. Since neurodegeneration is a crucial component of the pathogenic feature of AD and is not unique to the disease, parentheses are used for “(N)”.

Amyloid-β (Aβ) protein in extracellular plaques is the first pathology of AD that can be seen in the brain, and it manifests decades before clinical symptoms appear [8]. Neuronal degeneration, which is visible on structural MRI brain scans [9], and tau hyperphosphorylation, which causes neurofibrillary tangles [10], emerge next. In addition to offering a trustworthy means of diagnosing AD, biomarkers for amyloid and tau, neurodegeneration, and inflammatory alterations in cerebrospinal fluid (CSF) have given researchers a crucial basis of information for comprehending the earliest molecular alterations that take place in the disease [11]. Nevertheless, new ultra-sensitive plasma assays have made it possible to quantify A, T, and (N) accurately in blood samples, negating the necessity for lumbar punctures and lowering the amount of neuroimaging used in clinical and research settings [12].

The validation of the ATN biomarker framework led to methodological and conceptual advancements, which were acknowledged in the 2018 update of the NIA-AA research framework for the biological definition and diagnosis of AD [13]. The AT(N) framework’s methodological validation showed that it captures the essential elements of the AD pathology, spanning from the disease’s early stages to the dementia stage [14,15]. Conceptually, the AT(N) framework was found to not only represent the essential elements of the AD pathology but also to be unrelated to the clinical stage, meaning that the pathological basis is not always linked to any clinical outcomes. Core 1 biomarkers, such as α-amyloid Aβ42, phosphorylated tau, and amyloid PET, determine early AD phage detection and can identify AD in both symptomatic and asymptomatic individuals. Core 2 biomarkers, such as MTBR-tau243, p-tau205 non-phosphorylated mid-region tau fragments, and au PET, become abnormal later in the AD evolution and inform the short-term progression risk in people without symptoms [14]. The introduction of disease-modifying therapies and the expanding use of plasma biomarkers for both a clinical AD diagnosis and therapy response monitoring are reflected in the most current version of the NIA-AA criteria [14].

In this review, we will explore (a) the role of biomarkers in enhancing the diagnostic accuracy of AD, highlighting the major strides that have been made in recent years; (b) the role of neuropsychological testing in identifying biomarkers of AD, including the relationship between cognitive performance and neuroimaging biomarkers; (c) the amyloid hypothesis and possible molecular mechanisms of AD; and (d) the innovative AD therapeutics and the challenges and limitations of AD research.

## 2. The Role of Fluid Biomarkers in Early Diagnosis

### 2.1. Classical Neurodegenerative Biomarkers

The Aβ42/Aβ40 ratio is preferred as it is a superior diagnostic tool. Plasma Aβ42/Aβ40 levels are modified during the pre-symptomatic phase, allowing for the early detection of the Aβ pathology in cognitively normal subjects with comparable accuracies to cognitively abnormal people [16]. P-tau217, the strongest among p-tau markers, is a strong diagnostic tool and distinguishes AD from other dementias with a higher accuracy. The strongest diagnostic p-tau biomarker has been suggested to be P-tau217 (compared with p-tau181, p-tau231, and p-tau205). The area under the receiver operator characteristic curve (AUC), 0.943 vs. 0.914 and *p* = 0.026, indicates that CSF p-tau217 is a more effective diagnostic tool than p-tau181 [17]. Concurrently, CSF p-tau217 levels reliably differentiate AD from other dementias, outperforming p181. Plasma p-tau181 and p-tau217 accurately predict when MCI will progress to AD dementia in the future (between two and six years) [18,19]. Nevertheless, p-tau217 increases during the asymptomatic phase and changes as AD progresses, allowing for the early detection and prediction of AD, whereas higher p-tau217 levels suggest a rapid cognitive decline [20]. Regarding the T in the peripheral A-T-N-X framework, p-tau217 is a suitable biomarker given the aforementioned advantages.

**Figure 1:**
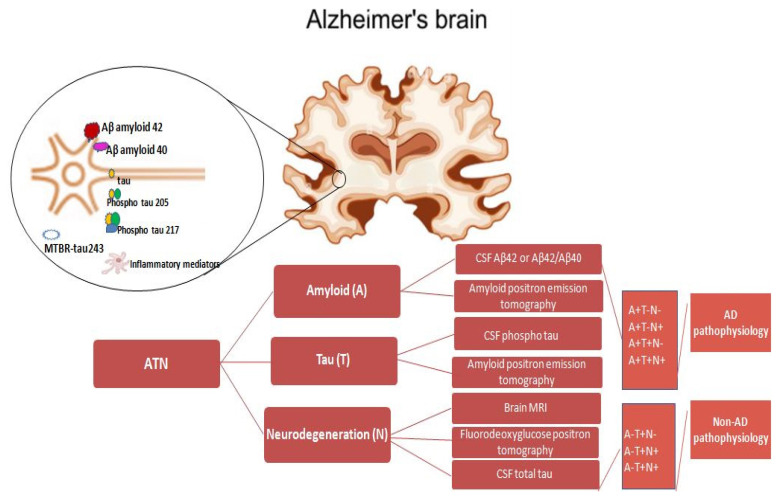
ATN profiles and corresponding biomarker categories. The biomarkers include neuroimaging and biofluids, primarily cerebrospinal fluid (CSF), and are categorized into beta-amyloid deposition (A), pathologic tau (T), and neurodegeneration (N), together referred to as the ATN criteria. The A^+^T^+^N^+^ biomarker profile supportive of AD does not exclude important comorbidities such as dementia with Lewy bodies, frontotemporal lobar degeneration, or non-AD neurodegeneration, such as limbic-predominant age-related TDP-43 encephalopathy (non-AD pathophysiology).

CSF p-tau231 is already significantly elevated before a definite Aβ pathology and is associated with an Aβ PET confinement in brain areas commonly impaired early on in AD [21]. While CSF p-tau231 prematurely stopped the Aβ alterations in the preclinical stage, CSF p-tau217 displayed the largest fold-change increases in disease-symptomatic phases. One important finding of this study is that, even in the absence of a clear Aβ pathology, CSF p-tau231 remains markedly raised. In cognitively unimpaired patients, CSF p-tau231 was strongly linked to an Aβ PET confinement in brain regions that are frequently affected early on in AD, such as the posterior cingulate cortices, precuneus, and medial orbitofrontal [22]. These biomarkers are useful in identifying the “AD neurochemical fingerprint” in atypical or mixed cases, as confirmed with PET imaging (Figure 1).

The A, T, and N research framework integrates biomarkers in clinical trials and drug development apart from integrating them into the AD diagnostic process (Table 1).

An immunoglobulin G1 monoclonal antibody called donanemab binds the shorter, insoluble form of β-amyloid that is present in brain amyloid plaques. By attaching itself to the β-amyloid’s N-terminally truncated form, it makes it easier for the microglia to phagocytose and eliminate plaque [23]. Changes in the plasma pTau217 and glial fibrillary acidic protein were significantly correlated with the centiloid percent change in amyloid following donanemab therapy. Reduced plasma levels of pTau217 and GFAP were linked to altered brain amyloid plaques detected by PET [24]. Donanemab delayed the rate of cognitive decline when compared to a placebo.

Lecanemab significantly increased CSF Aβ42 levels after 12 and 18 months, although Aβ40 concentrations remained unchanged [26]. The levels of CSF tTau, pTau181, and NRGN decreased during the 12- and 18-month follow-up. Lecanemab enhanced cognitive scores, reduced pTau181 and GFAP, and increased the Aβ42/40 ratio when compared to a placebo.

Phase 2 trials for semorinemab, gosuranemab, and tilavonemab (target class: Tau) are investigating monoclonal antibodies that do not exhibit any appreciable therapeutic effect [25,27,28,30,31,32].

Gosuranemab decreased CSF N-terminal tau, but semorinemab decreased CSF pTau181, pTau217, and tTau [31]. Neflamapimod, an inhibitor of p38α kinase, demonstrated a positive trend for NRGN and reduced CSF levels of tTau and pTau181 in comparison to a placebo [29].

### 2.2. Genetic Biomarkers

Genetic biomarkers play a crucial role in detecting people at risk for AD. The familial form of genetic AD is autosomal dominant, early-onset (EOAD) in people under 65, and typified by mutations in particular genes. Genetic testing for these variants helps identify people at risk for AD and informs personalized prevention and treatment strategies. Variants in the apolipoprotein E (APOE) gene have been strongly correlated with a high risk of AD, with the APOE ε4 allele being the most well-established genetic risk factor for late-onset AD. In the same line as APOE, recent genome-wide association studies (GWASs) reported over 30 genetic loci (CLU, PICALM, CR1, BIN1, EPHA1, MS4A, ABCA7, CD33, and CD2AP) associated with a late-onset AD risk, highlighting the polygenic nature of the disease [33]. The general population is frequently affected by sporadic AD, manifesting as late-onset AD (LOAD) in people over 65. The heritability of the condition can reach 60–80%. Rare variants (allele frequency) that influence the risk for LOAD have also been detected in several genes, including TREM2, PLD3, UNC5C, AKAP9, ADAM10, and ABI3.

The genetic basis for amyloid precursor protein profusion in Trisomy 21, also known as Down syndrome (DS), is EOAD. By the mid-40s, all DS patients have enough ADNPC to meet the neuropathological criteria for an AD diagnosis. Increased levels of peripheral proteins, including Aβ40; Aβ42; MMP-1, 3, and 9; proNFG; and inflammatory mediators like interferon gamma (IFN-γ), Tumor Necrosis Factor alpha (TNF-α), interleukin (IL)-6, IL-10, and IL-1, were among the changes in plasma biomarkers found in DS [34].

In addition to personalized treatment strategies, genetics can inform the development of personalized treatment strategies for AD patients based on their genetic profiles. Pharmacogenomic studies have identified genetic variants that influence individual responses to AD medications, and genes implicated in AD risk through GWASs can provide valuable insights into disease mechanisms and pathways that may be targeted for therapeutic interventions.

### 2.3. The Utility of Memory and Executive Function Tests in Predicting Disease Progression

One of the key challenges in AD research is predicting the progression from MCI to dementia and identifying individuals at higher risk of developing the disease. The Working Group suggested the Visual Short-Term Memory Binding Test (VSTMBT [35]) and the Free and Cued Selective Reminding Test (FCSRT [36])—two memory tests that have recently shown promise in the evaluation of preclinical AD. Parra et al. [37] recently suggested that the VSTMBT may need to be titrated to the targeted population (e.g., preclinical or prodromal) by adjusting the memory load (i.e., two or three items) in order to achieve the best classification power. Another memory test, the Rey Auditory Verbal Learning Test (RAVLT-LR), found that delayed recall was a significant predictor of the MCI-to-AD conversion within a 3-year period of follow-up [38]. These neuropsychological tools play a crucial role by allowing clinicians to detect subtle changes in cognitive abilities, such as memory impairment.

However, executive function tests have shown a low predictive accuracy for disease progression in AD with a moderate sensitivity and specificity [39]. Executive dysfunction is commonly observed in the early stages of the disease and has been associated with a greater cognitive decline and functional impairment over time. For example, the Clock Drawing Test, which assesses visuospatial and planning abilities, has been linked to the progression of AD and is predictive of future cognitive decline [35]. The Raven Colored Progressive Matrices test has been demonstrated to be an independent predictor of the conversion from MCI to AD at three years of follow-up. Finally, the Trail Making Tests A and B, in addition to attention and processing speed, proved to be useful in identifying the conversion to probable AD in elderly people with MCI within the next four years.

Furthermore, studies have demonstrated that a combination of memory and executive function tests can improve the accuracy of predicting AD progression compared to using either domain alone. For instance, a recent study found that a composite score of memory and executive function measures was highly predictive of dementia conversion in individuals with MCI, outperforming individual cognitive tests or neuroimaging biomarkers alone [40]. These findings highlight the importance of incorporating a comprehensive neuropsychological assessment in the early detection and prediction of the AD progression. By identifying cognitive deficits that are associated with specific neuropathological changes, clinicians can better target interventions and monitor the disease progression in at-risk individuals.

### 2.4. The Relationship Between Cognitive Performance and Neuroimaging Biomarkers

Neuroimaging techniques such as magnetic resonance imaging (MRI) and positron emission tomography (PET) can detect structural and functional changes in the brain that are associated with AD. These changes include atrophy in the hippocampus and other regions involved in memory formation, as well as the accumulation of amyloid plaques and neurofibrillary tangles, which are hallmark pathological features of the disease. Subtypes of atrophy in AD predict an early onset, shorter disease duration, and APOE ε4-positive patients. An MRI study by Persson et al. [41] showed four subtypes in 123 patients with mild AD, including “typical”, “limbic-predominant”, “hippocampal-sparing”, and “minimal atrophy”. The minimal atrophy subtype group was less educated, had greater functional impairment, and had higher levels of Aβ in cerebrospinal fluid. Cortical atrophy patterns correlate with cognitive impairment.

As the disease progresses, changes in cognitive function are strongly linked with the pace of change in anatomical markers such as the whole-brain, entorhinal cortex, and hippocampal volumes and ventricular enlargement [41]. For example, individuals with MCI or early-stage AD typically show a reduced performance on memory tasks such as the Rey Auditory Verbal Learning Test (RAVLT) or the California Verbal Learning Test (CVLT), which are sensitive to hippocampal dysfunction [42]. These memory deficits are often correlated with hippocampal atrophy measured on MRI or the increased amyloid deposition detected on PET scans. Similarly, executive function tests, such as the Trail Making Test or the Stroop Test, have been shown to be associated with patterns of the cortical thickness and white matter integrity in the prefrontal cortex, which are regions involved in executive control and decision-making. Nevertheless, amyloid pathology markers frequently exhibit more noticeable anomalies than structural markers during the shift from asymptomatic to MCI stages.

By integrating neuropsychological testing with neuroimaging biomarkers, researchers can gain a more comprehensive understanding of the cognitive and biological changes that occur in AD. This multimodal approach allows for the identification of specific cognitive profiles that are linked to distinct neuropathological features of the disease, thereby enhancing early detection and personalized treatment strategies.

### 2.5. Challenges and Limitations of Using Neuropsychological Testing in Biomarker Research

Neuropsychological testing is increasingly being used to identify biomarkers of AD, but there are several challenges and limitations to consider. The heterogeneity of cognitive profiles in AD can complicate the interpretation of these tests, as different individuals may exhibit varying patterns of deficits and abnormalities. A more targeted assessment of these cognitive domains may be necessary to capture the unique cognitive profile of each individual and its underlying neurobiological correlates. Additionally, there is a lack of standardized neuropsychological tests specifically designed to map onto specific biomarker changes in AD. A multidisciplinary approach incorporating neuroimaging, genetic, and biochemical assessments is essential for validating the cognitive–behavioral correlates of biomarker findings and establishing a comprehensive understanding of the disease process. The timing of neuropsychological testing in relation to biomarker measures is critical for capturing the dynamic changes that occur in AD. Longitudinal studies that track the cognitive performance and biomarker levels over time are needed to elucidate the temporal sequence of the cognitive decline and its relationship to neurobiological changes.

### 2.6. The Future of Biomarkers in AD

As our understanding of the molecular mechanisms underlying AD continues to evolve so too will the role of biomarkers in diagnosis and treatment. Future research efforts are likely to focus on the discovery of novel biomarkers that can provide insights into the early stages of the disease process, allowing for even earlier detection and intervention. For example, recent studies have shown that changes in the levels of microRNAs in the blood are associated with AD, suggesting that these small molecules may serve as promising new biomarkers for the disease. In addition to improving diagnostic accuracy, biomarkers also have the potential to revolutionize the development of new treatments for AD. By identifying individuals who are at high risk for developing the disease, researchers can enroll them in clinical trials of experimental therapies at the earliest stages of the disease, when interventions are most likely to be effective. This personalized medicine approach holds great promise for the future of Alzheimer’s research, offering the potential to tailor treatments to the specific molecular pathways that are driving the disease in each individual.

## 3. The Amyloid Hypothesis Is Receding

The amyloid hypothesis is predicated on the assumption that the extracellular accumulation of amyloid-β in the brain initiates a cascade of events, the most important of which is the formation of intracellular neurofibrillary tangles of hyperphosphorylated tau protein, and that this cascade ultimately causes nerve cell death [43,44]. Over the intervening years, drug development based on reducing the amyloid burden has yielded anti-amyloid immunotherapies with limited efficacy and a side effect profile that includes brain swelling and bleeding [45,46]. While anti-amyloid treatment is highly effective in transgenic murine models designed to overexpress amyloid, human AD cannot be reduced to this one-dimensional process, and the assessment of true disease-modifying properties is challenging [47,48,49]. Transgenic AD mice cannot replicate the human disease with fidelity for many reasons, including the oversimplification of the pathology when it is generated by changing a single gene or a few genes, as well as differences in the lifespan and brain structure [50].

Two recent phase 3 trials, each with close to 1000 participants with mild cognitive impairment due to AD, reported that the monoclonal antibody gantenerumab failed to show clinical benefits [25,51]. The reduction in amyloid plaque by PET scans confirms the removal of extracellular Aβ, but without the hoped-for abatement of symptoms. Hence, these repeated failures of Aβ-targeted clinical trials cast doubt on the amyloid cascade hypothesis and AD drug development targets [52,53]. Additionally, the weak correlation between the plaque load and cognition questions the role of Aβ plaques in vivo [54].

A number of explanations for the underwhelming outcomes of Aβ-targeting AD treatments have been proposed over the years. The most prominent reasons include missing the chance for immunoprevention due to the late initiation of therapy over the course of the AD development, inappropriate drug dosages, the wrong selection of treatment targets, and the neglect of the need for combination multidrug therapy to tackle the complex pathophysiologic mechanisms of AD [1,55,56]. High rates of drug discontinuation may also contribute to the problem.

The bias towards the amyloid hypothesis ignores other independent factors, such as genetic risks. These genetic risk factors impact the amyloid accumulation independently and dependently, as well as AD progression. Some notable genetic links are the APOE4 allele, familial mutations in presenilin1 (PSEN1), PSEN2, or the amyloid precursor protein (APP), and individuals affected with trisomy 21 [54,57,58]. The amyloid hypothesis is flawed in that it does not adequately consider these genetic components of AD and its multifactorial nature. The modest results of the Aβ-targeting treatment provide evidence that a one-size-fits-all approach is not tenable [59,60].

## 4. Molecular Mechanisms

**Figure 2:**
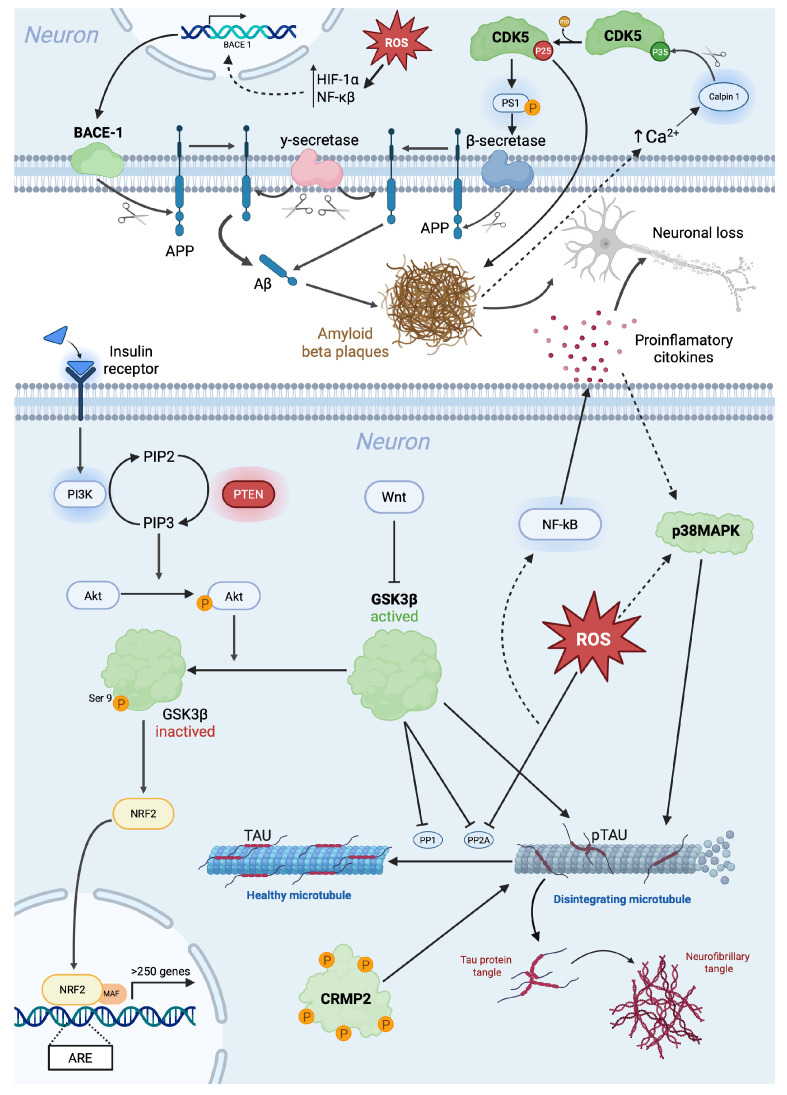
Schematic representation of molecular mechanisms involved in Alzheimer’s disease. Beta-site amyloid precursor protein cleaving enzyme (BACE); cyclin-dependent kinase 5 (Cdk5); collapsin response mediator protein-2 (CRMP2); glycogen synthase kinase 3 (GSK3); hypoxia-inducible factor 1-alpha (HIF-1α); phosphoinositide 3-kinase (PI3K); protein kinase B (Akt); reactive oxygen species (ROS); nuclear factor erythroid 2-related factor 2 (Nrf2); phosphatidylinositol 4,5-bisphosphate (PIP2); phosphatidylinositol-3,4,5-trisphosphate (PIP3); phosphatase and tensin homolog (PTEN); protein phosphatase 1 (PP1); protein phosphatase 2A (PP2A), soluble APP-beta (sAPPβ); and nuclear factor-kappa B (NF-κB).

The primary neuropathological hallmarks of AD include extracellular β-amyloid (Aβ) deposits forming amyloid plaques and intracellular accumulations of hyperphosphorylated tau protein within neurofibrillary tangles (NFTs) [61]. Moreover, AD is associated with vascular dysfunction [62], neuroinflammation [63], oxidative stress [64], and the dysregulation of the lipid and sphingolipid (SL) metabolism [65], among other pathological processes, implicating several molecular mechanisms (Figure 2).

### 4.1. BACE

The beta-site amyloid precursor protein cleaving enzyme (BACE), also known as beta-secretase, is a crucial protease involved in the amyloidogenic processing of the amyloid precursor protein (APP). This cleavage results in the production of Aβ peptides, which aggregate into amyloid plaques, a hallmark in AD [66]. Given its critical role in Aβ generation, the BACE has been extensively studied as a potential therapeutic target for AD.

The principal isoform of the BACE implicated in AD is BACE1, which is a transmembrane aspartyl protease predominantly expressed in neuronal cells. BACE1 contains an extracellular catalytic domain responsible for the initial cleavage of APP at the beta-site, forming the soluble APP-beta (sAPPβ) and the membrane-bound C-terminal fragment (CTF-β), which is further processed by γ-secretase to generate Aβ peptides [67]. Increased BACE1 expression and activity have been observed in the brains of AD patients, correlating with elevated Aβ production [68]. The resulting accumulation of amyloid plaques triggers a cascade of pathological events, including neuroinflammation, tau hyperphosphorylation, synaptic dysfunction, and neuronal loss [66].

A second isoform has been described with similar enzymatic properties, but its relationship to the AD pathology is unclear [69].

BACE1 activity is modulated by several intracellular and extracellular factors. There is a transcriptional regulation since BACE1 expression is influenced by transcription factors such as hypoxia-inducible factor 1-alpha (HIF-1α) and nuclear factor-kappa B (NF-κB), which are upregulated under oxidative stress conditions [70]. Moreover, there are some post-translational modifications, such as phosphorylation, ubiquitination, and glycosylation, affecting its stability and localization within cellular compartments [71]. Its lipid raft association is also important since BACE1 preferentially localizes to lipid rafts, specialized membrane microdomains that facilitate APP processing. Alterations in the lipid raft composition, as seen in AD, enhance the BACE1 cleavage activity [72].

BACE1 has been postulated as a therapeutic target; therefore, BACE1 inhibitors have been explored as a therapeutic strategy for AD. Several small-molecule inhibitors have been developed, such as verubecestat, lanabecestat, and atabecestat, which effectively reduce Aβ levels in preclinical models. However, clinical trials have largely failed due to adverse effects, including cognitive worsening, likely due to the physiological functions of BACE1 in synaptic plasticity and myelination [67,73]. Clinical trials have shown that BACE1 inhibitors effectively lower Aβ levels in cerebrospinal fluid and reduce the plaque burden in neuroimaging studies. However, significant adverse effects have been observed, including mild and reversible cognitive worsening, possibly due to the inhibition of the BACE’s role in processing other key neuronal proteins. The high-dose BACE inhibition (>50%) has been linked to these side effects, raising concerns about the viability of this approach. Verubecestat, a BACE inhibitor, demonstrated significant reductions in Aβ levels but failed to show cognitive benefits and was associated with cognitive decline in some patients, leading to trial discontinuation. Atabecestat and lanabecestat also reduced Aβ levels in cerebrospinal fluid (CSF) and plasma but faced safety concerns, such as liver toxicity and cognitive impairment [74]. Some studies suggest that partial inhibition (~30%) may be sufficient to slow AD progression while avoiding cognitive impairment [75].

Despite setbacks, future research is exploring lower-dose BACE1 inhibition as a preventive strategy for high-risk individuals or as a maintenance therapy following amyloid clearance with monoclonal antibodies. Understanding the precise mechanisms of cognitive side effects remains essential for optimizing BACE-targeted therapies [75].

Despite setbacks in the BACE1 inhibitor development, alternative approaches are being explored, such as partial inhibition strategies to maintain essential BACE1 functions while reducing pathological Aβ production [76]. Additionally, the modulation of BACE1 regulatory pathways, rather than direct enzymatic inhibition, may offer safer therapeutic options [74].

### 4.2. GSK3β: Glycogen Synthase Kinase 3-β

Glycogen synthase kinase 3 (GSK3) is a constitutively active and ubiquitously expressed proline-directed serine/threonine kinase that plays several roles in many physiological processes, from glycogen metabolism to gene transcription. There are two GSK3 genes from which GSK3α and GSK3β isoforms derive [77].

Multiple signaling pathways, primarily through phosphorylation events, tightly regulate GSK3 activity. The insulin and Wnt pathways act as major negative regulators of GSK3β. In the insulin/Phosphoinositide 3-kinase (PI3K)/Protein Kinase B (PKB/Akt) PI3K/AKT pathway, insulin binding to its receptor activates PI3K, which phosphorylates AKT. Activated AKT phosphorylates GSK3β at Ser9, leading to its inhibition and reducing its ability to phosphorylate downstream targets like tau. Phosphatase and the tensin homolog (PTEN), a phosphatase, negatively regulate PI3K, thereby enhancing GSK3β activity. In the Wnt pathway, Wnt signaling leads to the inactivation of GSK3β by sequestering it in a protein complex, preventing β-catenin degradation and promoting cell survival [78,79].

In the context of AD, GSK3, more exactly GSK3-β, has been proposed as a central mediator of pathogenesis and plays a leading role in the cascade of events that culminate in AD, such as the hyper-phosphorylation of tau, the increased production of Aβ, memory impairment, and neuroinflammation. Therefore, it is related to almost all the hallmarks of AD [78]. Several studies argue that the increase in the expression or activity of Ser/Thr kinases such as CDK5 or GSK3β decreases the expression of Protein Phosphatase 1 (PP1) and Protein Phosphatase 2A (PP2A), which are phosphatases whose function is to dephosphorylate tau [80,81]. Moreover, it has been observed in specific brain regions, principally the hippocampus, in AD patients that with the increase in GSK3β, there is a decrease in PP2A activity [82,83].

GSK3β has been described as a key kinase involved in the hyperphosphorylation of tau, leading to the formation of neurofibrillary tangles (NFTs) [84]. GSK3 is a kinase of significant importance for p-tau, with approximately 30 serine or tyrosine residues from tau identified as potential phosphorylation sites for GSK3 [85]. Some studies suggest that GSK3β is responsible for the direct phosphorylation of tau at multiple sites, which promotes its aggregation into filamentous structures resembling those observed in AD brains. Studies have observed an increase in the GSK3 expression in AD patients’ brains and models [86].

In this case, PI3K/AKT-mediated GSK3 signaling is important in the tau pathology. The PI3K/AKT/GSK3β pathway is crucial in tau phosphorylation, with AKT inhibiting GSK3β to maintain tau stability [87]. However, neuroinflammation disrupts this balance, as C-reactive protein and IL-1β enhance tau phosphorylation via AKT/GSK3β modulation [88]. Additionally, H_2_S has dual effects: while IL-1β-induced H_2_S impairs AKT’s inhibition of GSK3β, leading to tau hyperphosphorylation, exogenous H_2_S can directly inhibit GSK3β and reduce the tau pathology [89]. GSK3β also interacts with caspase-3, which cleaves AKT, further increasing tau phosphorylation [90]. The insulin signaling impairment exacerbates this, with the REG-1α overexpression and NIR knockout promoting the tau pathology [91]. In contrast, PLTP and mGluRs modulate PI3K/AKT, reducing the GSK3β activity and tau hyperphosphorylation [92].

Adenosine Monophosphate-Activated Protein Kinase (AMPK) plays a key role in the bioenergy metabolism and AD, with a reduced activity observed in AD models. Increasing the AMPK expression alleviates tau hyperphosphorylation, while GSK3β agonists like wortmannin counteract this effect, highlighting GSK3β’s role in AMPK-mediated tau suppression [93]. Additionally, Cannabinoid Receptor 2 (CB2R) activation limits GSK3β activity and tau phosphorylation, but this protection is lost if AMPK is inhibited. Adiponectin promotes AMPK activation, leading to GSK3β inhibition and a reduced tau pathology, though cerebral hypoglycemia may induce tau phosphorylation via the same pathway [94,95].

GSK3 has been demonstrated to regulate the tau pathology through signaling mediated by other molecules, such as proteins, miRNA, and micromolecules [96,97,98].

In AD investigations, an important field has been the investigation of the correlation between the aggregation of Aβ and tau. GSK3-β has appeared as a crucial element in receiving Aβ stimuli and promoting the tau pathology. Aβ can promote tau hyperphosphorylation by activating tau kinases like GSK3β [99,100]. Additionally, Aβ-induced inflammation may further contribute to the tau pathology. As a key driver of innate immune activation, Aβ triggers inflammatory responses and stimulates the release of pro-inflammatory cytokines, such as IL-1β [101,102].

GSK3β plays a crucial role in the amyloid pathology by influencing APP processing, β-secretase (BACE1), and γ-secretase activity, ultimately affecting Aβ production [103,104]. GSK3β activation has been linked to an increased amyloid plaque formation in AD models, where its inhibition reduces the Aβ accumulation in the cortex and hippocampus [105]. Conversely, β-secretase inhibition can paradoxically activate GSK3β, intensifying tau hyperphosphorylation [106].

Aging models like SAMP8 mice show PI3K/AKT pathway dysregulation, leading to GSK3β activation and increased Aβ1-40/Aβ1-42 levels [107]. Additionally, RAGE signaling has been identified as a bridge between GSK3β and the amyloid pathology, with its inhibition reducing the Aβ accumulation and β-/γ-secretase activity [108]. The Wnt/β-catenin pathway also plays a role, as the GSK3β inhibition enhances Wnt signaling and suppresses BACE1 transcription [109].

GSK3β further regulates APP transcription, phosphorylation, and degradation, contributing to the amyloid burden [110]. Astrocytes can transmit CK1 to neurons via extracellular vesicles, forming a CK1–APC–GSK3 complex that stabilizes β-catenin, upregulating the APP and BACE1 expression, thereby promoting Aβ generation [111]. Notably, the GSK3β inhibition reduces APP phosphorylation, alters cleavage processes, and enhances the autophagic degradation of APP via TFEB activation, ultimately lowering Aβ levels [112,113]. Furthermore, GSK3β inhibition protects hippocampal networks from Aβ-induced dysfunction, highlighting its role in Aβ sensitivity [114].

GSK3β plays a key role in oxidative stress and neuroinflammation, primarily through its regulation of nuclear factor erythroid 2-related factor 2 (Nrf2), a transcription factor responsible for antioxidant defense. However, GSK3β-mediated phosphorylation at Tyr216 enhances Nrf2 ubiquitination and degradation via the β-TrCP-Cul1 complex, suppressing its protective effects. The inhibition of GSK3β has been shown to enhance Nrf2 activity, improving the oxidative resilience and cognitive function in AD models [115].

GSK3β is also implicated in neuroinflammation, where its interaction with NF-κB, a central regulator of inflammatory responses, exacerbates neurodegenerative processes. Excessive reactive oxygen species (ROS) production facilitates NF-κB activation, driving inflammation through microglial and astrocytic responses. In AD, the activated microglia and astrocytes release inflammatory cytokines such as IL-1β, TNF-α, and iNOS, which further amplify neuronal damage. The GSK3β/Wnt pathway plays a critical role in this process by inhibiting PI3K/AKT, thereby maintaining GSK3β in an active state and promoting microglial activation [116,117].

Notably, modulating the GSK3β activity has shown promise in reversing AD-associated inflammation. For example, DHCR24 overexpression in microglial cells shifts the polarization toward an anti-inflammatory M2 phenotype, increasing IL-4 and TGF-β while reducing IL-1β and TNF-α levels. Similarly, the inhibition of KCa3.1, a calcium-activated potassium channel, has been associated with PI3K/AKT activation, GSK3β suppression, and NF-κB inhibition, leading to neuroprotection and cognitive improvements in AD models. These findings highlight the potential of GSK3β inhibitors as therapeutic agents targeting oxidative stress and neuroinflammation in AD [118,119].

Autophagy helps clear Aβ and p-tau through the lysosomal pathway, with mTOR, regulated by GSK3β, playing a key role in its activation [120]. DHCR24, previously mentioned, knockdown leads to GSK3β overactivation, which inhibits autophagy by phosphorylating mTOR at Ser2448, reducing autophagosome formation and exacerbating the AD pathology [121].

Some studies have shown that inhibiting GSK3β, specifically p-GSK3βSer9, benefits long-term memory formation [122]. This fact also positively correlates with studies that demonstrate that elevated GSK3β activity in the peripheral blood of AD patients correlates with dementia severity [123]. In this regard, the overexpression of GSK3β can inhibit synaptogenesis, a crucial process for memory processes [124]. Moreover, in AD mouse models exposed to Aβ oligomers, excessive GSK3β activation is associated with dendritic spine loss, further contributing to neurodegeneration [125]. Moreover, GSK3β’s role in the Wnt/β-catenin pathway exacerbates AD-related neuronal damage, given the pathway’s crucial function in synaptic plasticity and memory [126].

### 4.3. PP2A

Protein Phosphatase 2A (PP2A) is a large family of serine/threonine phosphatases highly expressed in the brain, and its malfunction has been linked to human disorders such as neurodegenerative diseases. PP2A and GSK-3β are key regulators of tau phosphorylation. Under normal physiological conditions, PP2A dephosphorylates tau, preventing its aggregation.

PP2A plays a pivotal role in maintaining tau protein homeostasis, and several studies have demonstrated that PP2A dysfunction plays a central role in the progression of the tau pathology in AD [127,128]. In addition, an altered PP2A expression has been described in AD autopsy brain tissues [129].

PP2A dysfunction has been associated with tau hyperphosphorylation, amyloidogenesis, and synaptic deficits, key pathological hallmarks of this neurodegenerative disorder. Additionally, PP2A deregulation impacts the activity of multiple Ser/Thr protein kinases involved in AD [127]. It has been described that, in the context of AD, PP2A/Bα holoenzymes are capable of binding directly to the microtubule-associated tau protein [127]. PP2A is one of the primary phosphatases responsible for dephosphorylating tau, which plays an essential role in neuronal homeostasis [130].

It is established that oxidative stress leads to the generation of reactive oxygen species (ROS), which damage proteins, lipids, and DNA, ultimately leading to neuronal dysfunction [131].

In this regard, several studies have pointed out that oxidative stress in the AD pathology inhibits PP2A while activating GSK-3β, leading to excessive tau phosphorylation and neurotoxicity. This imbalance between the PP2A and GSK-3β activity is a hallmark of the AD pathology [132,133]. Other studies have shown that ROS inhibits PP2A activity and consequently the overactivation of GSK3β, leading to the hyperphosphorylation of the tau protein and subsequent neurofibrillary tangle formation. In addition, recently it was observed that oxidative stress promotes the dissociation of the PP2A holoenzyme, reducing its functional capacity and impairing cellular homeostasis [134,135].

PP2A also modulates neuroinflammation through its regulation of nuclear factor-kappa B (NF-κB). In AD, ROS-induced PP2A inhibition results in the activation of NF-κB, which promotes the expression of pro-inflammatory cytokines. This inflammatory response further contributes to oxidative damage and neuronal loss, creating a vicious cycle of neurodegeneration [136].

Studies in rodent models with memory deficits have demonstrated that inhibiting PP2A with okadaic acid (OKA) induces tau hyperphosphorylation. Both in vitro and in vivo research suggests that oxidative stress deactivates PP1 and PP2A, leading to sustained ERK1/2 phosphorylation, which contributes to the formation of neurofibrillary tangles. Reduced PP2A activity further exacerbates the tau pathology by promoting the activation of ERK1/2, MEK1/2, and p70 S6 kinase, while impairing tau dephosphorylation [137].

Furthermore, PP2A counteracts the activity of several brain protein kinases that are upregulated in AD. Consequently, developing PP2A-targeted therapies, particularly against the P-tau pathology, could be highly impactful in treating the disease [138].

### 4.4. p38 MAPK

P38 MAPK is a class of protein kinases activated through a mitogen-mediated signaling cascade (MAPK). This protein responds to stress stimuli such as inflammatory cytokines and reactive oxygen species (ROS). Once activated, p38 MAPK phosphorylates various substrates such as regulatory proteins and transcription factors. Activated PKR can also control tau synthesis and induce its phosphorylation [139].

In vivo studies in transgenic mice with tau hyperphosphorylation have shown a positive correlation between p38 MAPK activation and the amount of aggregated tau [140]. In addition, Pei et al. showed that p38 MAPK activity is elevated in the cortex and hippocampus of both AD mice and patients, at relatively early stages of disease progression [141]. Furthermore, glial cells showed an overexpression of p38 MAPK stimulating the chronic release of inflammatory cytokines in astrocytes and microglia [142,143].

In particular, p38 MAPK can directly phosphorylate tau on multiple residues, promoting its aggregation and dysfunction [85]. This tau phosphorylation also occurs at specific residues associated with AD, such as Ser202, Ser396, Thr205, and Thr231, among others [144]. Furthermore, the frequent colocalization of activated p38 MAPK and hyperphosphorylated tau has been observed in neurons from the brains of AD patients [145]. On the other hand, p38 MAPK can activate the kinases GSK-3β and CDK5 by phosphorylating them, which, as previously mentioned, are directly related to tau phosphorylation.

RNA-activated protein kinase is required to activate p38 MAPK (PKR) [146]. Interestingly, aggregated Aβ can induce PKR activation [147]. Moreover, activated PKR was found to be elevated in the brains and CSF of AD patients [148,149]. Furthermore, the genetic blockade of PKR in the 5xFAD mouse model of AD has been shown to reduce the cognitive impairment, neuroinflammation, neurodegeneration, and Aβ 1-42 accumulation in the brains of these animals [150].

### 4.5. Cdk5

Cdk5 is a serine/threonine protein kinase that belongs to the cyclin-dependent kinase family. It is expressed in neuronal and non-neuronal cells; however, it exhibits a high activity in neuronal tissue [151]. Cdk5 is essential for neuronal development, maturation, and function, as it phosphorylates the specific serine or threonine sites of numerous substrates closely associated with these processes [152]. Cdk5 requires binding to its activator p35 to carry out its different functions, such as neuronal migration and differentiation, synaptic growth and functions, neurotransmission, gliogenesis, associative learning and long-term behavioral changes, retrograde axonal transport, the formation of the cortex layer, development, and normal functions of the cerebellum [153,154,155,156]. An increase in the Ca^2+^ concentration can induce the cleavage of p35 into p25 and p10 by calpain. Pathological conditions that induce an increase in the Ca^2+^ concentration can activate calpain. Upon activation, p35 cleavage may occur, generating p25, which is also able to stimulate Cdk5 activity, leading to a “hyperactive” kinase. Moreover, the p25/Cdk5 complex induces several pathological processes, such as tau hyperphosphorylation, Aβ formation, neuronal cell apoptosis, mitochondrial dysfunction, cell cycle reactivation, and oxidative stress. Specifically, p25 accumulates have been described in the brains of AD patients, supporting the idea that the dysregulated Cdk5 activity due to p25 accumulation may contribute to the pathogenesis of AD [157]. Furthermore, studies with mouse models overexpressing p25 observed neurodegeneration [158]. Additionally, Currais et al. showed that decreasing p25 levels in mice prevents the development of learning and memory deficits [159]. Specifically, the hyperactivation of Cdk5 is implicated in the early stages of AD, as Cdk5 is linked to aberrant APP phosphorylation, which influences Aβ formation [160]. Cdk5 phosphorylates APP at the Thr668 residue; this can modulate its processing, promoting Aβ production and reducing APP’s interaction with Fe65, a cytoplasmic protein that can inhibit Aβ generation [161].

Cdk5 can also directly phosphorylate PS1 at Thr354 and promote presenilin levels, thereby increasing β-secretase activity, which can increase the production of Aβ peptides [162]. In turn, Aβ accumulation increases intraneuronal calcium concentrations, which activates calpain, thereby generating p25 that hyperactivates Cdk5 [163]. Therefore, Cdk5 and Aβ form a positive feedback loop that induces the pathological events of AD.

On the other hand, Cdk5 is also closely related to the phosphorylation of tau protein and the production of neurofibrillary tangles [164]. Cdk5 physiologically phosphorylates many tau epitopes that appear hyperphosphorylated in AD brains [165,166]. In an AD mice model, Cdk5 inhibition reduced the number of neurofibrillary tangles in the hippocampus [167]. In neuronal cultures, tau hyperphosphorylation can be prevented by preventing the cleavage of p35 to p25 or by blocking the Cdk5 activity [167,168].

Cdk5 dysfunction also plays a role in synaptic plasticity, as the cAMP signaling is altered by the increased phosphodiesterase expression, resulting in impaired synaptic plasticity and hippocampal-dependent memory formation [169].

### 4.6. CRMP2

Collapsin response mediator protein-2 (CRMP2) is involved in the assembly of neuronal microtubules [170]. Its hyperphosphorylation reduces its ability to stabilize microtubules, as it cannot bind to them effectively, leading to the disorganization of the neuronal cytoskeleton and the loss of axonal integrity [171,172].

CRMP2 phosphorylation appears elevated in both human AD brains and animal models presenting with the disease [173,174]. Furthermore, the phosphorylated form of CRMP2 is also found in neurofibrillary tangles [175]. In AD-expressing mice, increased CRMP2 phosphorylation occurs before the onset of the pathology, indicating that CRMP2 hyperphosphorylation may be a very early process in AD [176]. It occurs before tau phosphorylation in AD animal models and brains [176]. Moreover, in AD mice, CRMP2 phosphorylation at Ser522 may also promote Aβ-induced tau phosphorylation [177]. Interestingly, Cdk5 and GSK3β also regulate the function of this through phosphorylation [171,178].

### 4.7. DKK1 and the Wnt Pathway

The extracellular protein Dickkopf-1 (DKK1) is an endogenous antagonist of the canonical Wnt pathway that is overexpressed in AD, in the brain, plasma, and CSF [179]. The inhibition of this signaling pathway is associated with the activation of GSK3β, which also leads to tau hyperphosphorylation, neurofibrillary tangle formation, and neuronal death [180]. In this regard, other studies have found that Wnt/β-catenin signaling is able to inhibit the amyloidogenic processing of APP by suppressing the transcription of the β-site APP cleavage enzyme [109,181].

## 5. The Delivery of Therapeutics Across the BBB

Addressing the central nervous system (CNS) to treat its disorders continues to present significant challenges, primarily due to the restrictive properties of the blood–brain barrier (BBB). The BBB is a highly specialized structure that tightly regulates the transport of molecules between the bloodstream and the brain. It effectively inhibits the passage of approximately 98% of small-molecule drugs and nearly 100% of large-molecule therapeutics [182]. Additionally, achieving target therapeutic actions exclusively within the brain is hindered by physical barriers and the widespread expression of drug targets in peripheral tissues. This often results in systemic drug distribution, leading to off-target accumulation and associated toxicities [183].

**Figure 3:**
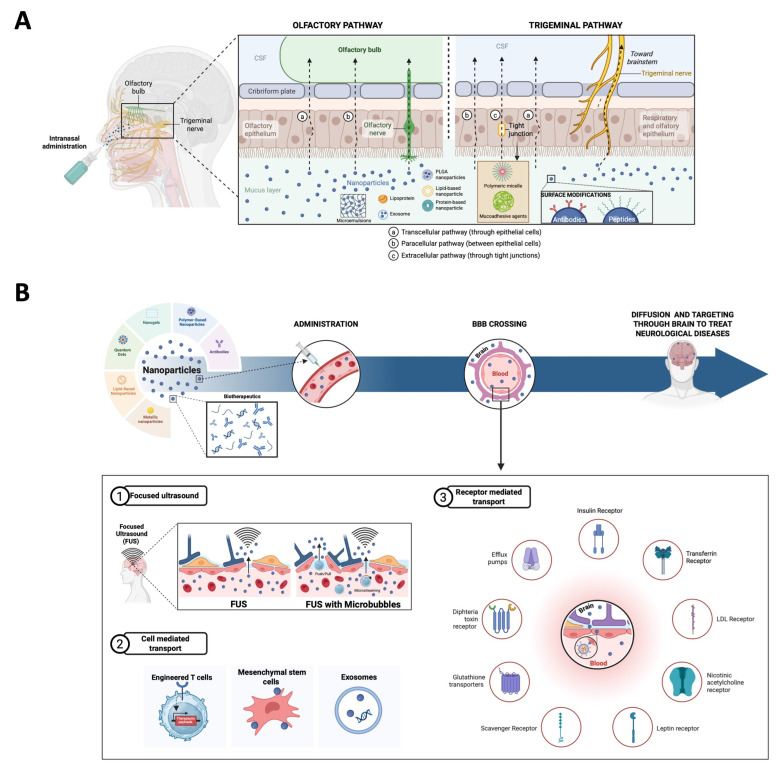
A schematic representation of therapeutic strategies to cross the BBB. (**A**) Intranasal administration. CSF—cerebrospinal fluid; PLGA—poly(lactic-co-glycolic acid. (**B**) Therapeutic strategies to directly cross the BBB. FUSs—focused ultrasounds.

In recent years, significant advancements in drug delivery technologies have aimed at overcoming this barrier to enhance the treatment efficacy for CNS disorders (Figure 3). Efforts have principally focused on modifying the physical or chemical properties of drugs to increase brain penetration, enhancing the BBB permeability or delivering therapeutics directly into the brain [184,185].

### 5.1. Intranasal Brain Delivery

Intranasal drug delivery to the CNS is a mechanism of BBB bypass through the trigeminal and olfactory nerves. This approach is an appealing alternative to conventional parenteral and oral routes, as it increases the drug concentration in the brain, ensures a rapid onset action by avoiding the hepatic first-pass metabolism, and offers a patient-friendly method of administration because it does not require sterile delivery and is painless [186,187]. Intranasal drugs have been demonstrated to reach the brain within 5 min and to reach the more remote areas of the brain in 30 min [188]. Two primary pathways have been described: olfactory and trigeminal pathways. In the olfactory pathway, drugs are transported via the olfactory nerve to the olfactory bulb directly. Molecules can cross through transcellular and paracellular routes depending on their lipophilicity or hydrophilicity, respectively. In the trigeminal pathway, the trigeminal nerve, which is divided into the ophthalmic, maxillary, and mandibular branches, transports drugs from the nasal epithelium to the brainstem via intracellular and extracellular mechanisms [189,190]. Besides these two pathways, there are some minor pathways such as vascular, CSF, and lymphatic pathways that, while less prominent, contribute to drug delivery through systemic absorption, allowing drugs to cross the BBB indirectly [191].

To enhance nasal permeation, there are two main options: permeation enhancers and mucoadhesive agents. Permeation enhancers are low-molecular-weight and biocompatible substances that improve drug transport by disrupting lipid bilayers, enhancing membrane fluidity and reducing the mucociliary clearance. Some examples are surfactants, fatty acids, or glycols [192,193]. Interestingly, a recent study using this strategy demonstrated that a nasal Donepezil-loaded microemulsion could be a potential tool for AD treatment [192]. Moreover, mucoadhesive agents are substances that increase the retention time of drugs in the nasal cavity by interacting with mucus and opening tight junctions. There are even some mucoadhesive thermosensitive gels, such as rivastigmine-loaded gels, that have demonstrated prolonged drug retention and enhanced absorption [194].

This strategy leverages nanocarriers and modulating agents to enhance the bioavailability and efficacy of therapeutic compounds, addressing the limitations of conventional delivery methods [195].

Nanoparticles play a crucial role as the nanocarriers, enhancing the efficacy of brain delivery. This is particularly important given the numerous limitations of intranasal delivery, such as the restricted dosage capacity, limited to under 200 μL; the rapid drug clearance in the nasal cavity due to the enzymatic activity (proteases and aminopeptidases); and the inherent anatomical barriers, such as the nasal mucus, epithelium, and lamina propria [196,197,198]. The main disadvantages related to nose-to-brain delivery include drug expulsion due to sneezing, the entry of drug molecules into the respiratory tract, and the irritation of the nasal cavity [196]. Inside the nanoparticles, surface modifications, such as esterification and surfactant methods, improve the stability and targeting efficiency of nanoparticles, like poly(lactic-co-glycolic acid) (PLGA) nanoparticles. Furthermore, ligands such as antibodies and peptides conjugated to nanoparticles bind specific receptors on target cells, enhancing drug efficacy, and this technique is called active targeting. Another option is passive targeting where drugs are encapsulated in polymer-based carriers that preferentially accumulate in diseased tissues [199,200].

On the other hand, in situ gels (ISGs) have emerged as a promising strategy to overcome challenges associated with nose-to-brain drug delivery. ISGs are clear or low-viscosity liquids that upon exposure to nasal physiological conditions, such as the pH, temperature, or ionic changes, transition into a viscous gel. This transformation extends the drug retention time in the nasal cavity, minimizes rapid drug elimination, and reduces the dosing frequency. ISGs have demonstrated an enhanced potential for controlled drug delivery directly to the brain via the intranasal route [186].

### 5.2. Focused Ultrasound

A focused low ultrasound represents a transformative approach to overcome the issue of delivery through the BBB in treating neurological disorders. Ongoing research indeed continues to refine this technology, aiming to enhance its efficacy, safety, and applicability across a broader spectrum of clinical scenarios.

#### 5.2.1. With Microbubbles

Focused ultrasound (FUS) has emerged as a promising non-invasive technique for enhancing drug delivery to the brain. By transiently disrupting the BBB, FUS allows the targeted delivery of therapeutics to the CNS. This method employs low-intensity focused ultrasound (LIFU) waves in conjunction with microbubbles to temporarily increase the BBB permeability; these openings usually close within 24 h [201].

Mechanistically, the procedure consists of administering microbubbles intravenously, which circulate to the cerebral vasculature. Using MRI to guide the ultrasound, it is possible to expose limited areas to low-intensity focused ultrasound waves, causing the oscillation of the microbubbles, leading to mechanical effects that temporarily disrupt the tight junctions of the BBB, enabling targeted opening of precise brain regions to perform the drug delivery. The use of transcranial MRI-guided focused ultrasound (MRIgFUS) combined with microbubbles causes no apparent damage to tissues or even long-term neurological effects. Nevertheless, work to optimize FUS protocols in humans continues [202,203]. The safety and feasibility of FUS-induced BBB opening are important considerations. Despite the ongoing advancements in MRIgFUS, it needs further research into monitoring techniques to ensure precise drug targeting and minimize potential adverse effects [204]. There are several advances in the field, like a novel ultrasound modality called Equivalent Time Active Cavitation Imaging, which has been developed to characterize the ultrasound pressure field during the treatment, which provides real-time monitoring to ensure both the effectiveness and the security of the BBB disruption [205].

FUS has been widely studied in clinical trials to evaluate its safety, tolerability, and feasibility, being one of the most prevalent techniques in recent clinical trials [182]. Low-intensity ultrasound waves have been approved by the FDA as a treatment for neurological disorders, such as essential tremor and tremor-dominant Parkinson’s disease [206]. This technique is currently under investigation for its potential applications in epilepsy [207] and neuropathic pain [208].

Extensive preclinical research supports the use of this intervention for the treatment of neurodegenerative diseases. Notably, opening the BBB without the use of therapeutics has been demonstrated to activate microglia, promote neurogenesis, clear amyloid-beta (Aβ) plaques in targeted regions, restore memory functions, and enhance the synaptic long-term potentiation [209]. A paper by Rezai et al. published in 2024 demonstrates that LIFU guided by MRI could reversibly open the BBB, enhancing the delivery of a monoclonal antibody targeting amyloid-beta plaques (Aducanumab) in patients with AD [210]. Studies in mice have further revealed that aging and the amyloid pathology amplify the BBB opening and prolong the time before its closure [211]. In mice models of AD and PD, a FUS-mediated BBB disruption has been shown to enhance the brain’s concentration of amyloid and tau antibodies, neurotrophic factors, and GSK-3 inhibitors [212,213,214].

#### 5.2.2. Without Microbubbles

FUS without microbubbles could be an interesting therapeutic strategy for neurodegenerative diseases due to its potential for neuromodulation. Recent research has been exploring the possibility of using Targeted Low-Intensity Ultrasound without microbubbles as a non-invasive approach for the neuromodulation of deep brain structures, bypassing the critical need for the BBB disruption. The preclinical studies have demonstrated its potential to increase the neural activity and cerebral blood flow and improve the neural functional connectivity [215]. There is even an open-label trial currently underway, enrolling 100 patients with PD or AD with mild cognitive impairment (MCI) or dementia. The patients of the study received transcranial-focused ultrasound targeting the putamen and substantia nigra for PD and the hippocampus for AD. The preliminary data indicates that the procedure may offer cognitive benefits, and it is safe [216].

### 5.3. Cell-Mediated Transport

#### 5.3.1. T Cells

T cells are emerging as a groundbreaking therapy for treating CNS diseases, primarily due to their ability to cross the BBB and deliver highly targeted treatments.

Simic et al. and their collaborators have designed T cells that can recognize endogenous CNS-specific antigens, more specifically in the ECM, using a synNotch receptor (engineered receptors designed to sense an extracellular antigen and trigger a transcriptional response) [217] to induce the production of therapeutic payloads locally and specifically in the brain, minimizing the systemic off-target effects like widespread immunosuppression or toxicity. They have used the BCAN (brevican) antigen that can selectively infiltrate the CNS for precise targeting. It is dual-level targeting because of the anatomically restricted activation (CNS) and the localized therapeutic delivery due to the molecular targeting specificity of the payload. These engineered T cells offer an adaptable platform to address a wide range of CNS disorders, from tumors to neurodegenerative or neuroinflammatory diseases [218].

#### 5.3.2. Stem Cells for Brain Drug Delivery

Mesenchymal stem cells (MSCs), including those derived from bone marrow, are believed to have the ability to cross the BBB, making them a promising vehicle for delivering therapeutics to the brain. MSCs can home to injured brain areas and cross the BBB through fenestrations or receptor-mediated mechanisms. They act as dynamic carriers for drugs, proteins, or nanoparticles, reducing off-target effects and addressing the challenge of the BBB impermeability. Engineered MSCs delivered therapeutic agents like dopamine, IL-10, or specific miRNAs, showing promise in Parkinson’s and AD models. MSCs can be modified to carry nanoparticles, improving drug delivery efficacy. Intranasal or carotid artery administration is suggested to bypass the pulmonary first-pass effect. Addressing immune compatibility for allogeneic MSCs remains crucial. MSCs represent a promising approach for the non-invasive, targeted delivery of therapeutics to the brain, addressing various neurological conditions [219].

#### 5.3.3. Exosomes for Brain Drug Delivery

Exosomes are nanoscale extracellular vesicles that naturally traverse the BBB and act as efficient drug delivery vehicles due to their biocompatibility, low toxicity, and ability to carry both hydrophobic and hydrophilic drugs. Exosomes use receptor-mediated transcytosis and endocytosis facilitated by BBB-specific receptors like transferrin and GLUTs. They achieve transcellular passage via multivesicular body (MVB) pathways and endothelial interactions. Their surface proteins and inherited contents from parent cells make them ideal for targeting and intercellular communication in the brain. Exosomes loaded with therapeutic agents, such as siRNA or enzymes, show promise for conditions like Alzheimer’s and Parkinson’s by reducing neurotoxicity and oxidative stress [220]. Therefore, exosomes are a potential and versatile non-invasive approach for drug delivery across the BBB.

### 5.4. Receptor-Mediated Transporters at the BBB

To overcome the limited paracellular transport rate across the blood–brain barrier (BBB) and meet the brain’s metabolic needs, the endothelial cells of the BBB must express specific receptors and/or transporters. These are essential for facilitating the passage of vital molecules, including nutrients, neurotransmitters, and amino acids, across the barrier [221]. Using these receptors, engineered nanotechnologies could reach the BBB.

#### 5.4.1. Insulin Receptor

The insulin receptor (InsR) is expressed on the luminal side of BBB endothelial cells, and when it interacts with the insulin ligand it can trigger two processes: the receptor-mediated transcytosis, allowing insulin to move from the bloodstream to the brain, or the activation of a signaling cascade that promotes brain metabolic activities. This activation can be stimulated not only by insulin but also by other receptor agonists, such as insulin-like growth factor 1. The insulin receptor (InsR) and the insulin-like growth factor receptor (IGF-R) are similar, but the IGFR has a higher affinity for insulin-like growth factors [222].

The direct use of insulin as a targeting ligand for the InsR is not feasible due to its very short half-life and the potential for side effects associated with its biological activity. Similarly, using an antibody targeting the InsR can also lead to adverse effects, as it may cause direct competition for the ligand-binding site [223,224].

#### 5.4.2. Transferrin Receptor

This is likely the most studied and widely exploited receptor expressed at the BBB. Each subunit of this receptor has the ability to bind to a single transferrin (Tf) molecule, which is the protein responsible for binding and transporting iron throughout the human body.

Studies in Rhesus monkeys confirm that high-affinity anti-TfR1 antibodies achieve a significant CSF distribution post-intravenous administration. These findings highlight the potential of TfR1 as a therapeutic target for delivering drugs and diagnostics across the BBB and into the brain parenchyma or CSF [225].

It has been demonstrated that transferrin receptor (TfR) levels and TfR-mediated internalization mechanisms at the blood–brain barrier (BBB) are preserved in the presence of the AD neuropathology, including Aβ and TAU pathologies [226].

#### 5.4.3. LDL Receptor

The primary role of the low-density lipoprotein receptor (LDLR) at the BBB is to regulate cholesterol homeostasis by mediating the internalization of cholesterol-rich low-density lipoproteins (LDLs), such as apolipoprotein B and E. While this process is much more prominent in the liver, it also takes place at the BBB [227]. At the BBB, the LDLR is expressed not only by brain endothelial cells but also by astrocytes. Additionally, astrocytes have been shown to regulate the LDLR expression on endothelial cells by releasing soluble factors. The use of LDLR ligands has not been linked to significant off-target effects or competition for receptor-binding sites. As a result, apolipoproteins B and E are effective targeting agents and have been utilized as ligands in BBB-penetrating delivery systems. Additionally, an LDLR-targeting peptide, Angiopep-2, has shown promising results as a receptor-mediated transcytosis (RMT) mediator, outperforming other candidates [228,229,230].

#### 5.4.4. Nicotinic Acetylcholine Receptor

The primary physiological ligand of the nicotinic acetylcholine receptor (nAchR) is the neurotransmitter acetylcholine; however, it has been demonstrated that the alkaloid nicotine can also effectively target this receptor. The nAchR is highly expressed in the central nervous system, reflecting its critical role in modulating acetylcholine transport [231,232].

The exact mechanism behind the receptor remains unclear; however, certain targeting ligands—such as RVG-29, derived from the rabies virus glycoprotein—have shown promise in achieving selective brain targeting. Similarly, ligands like (D)CDX have proven effective in targeting the nAchR and enabling transport across the BBB [233].

#### 5.4.5. Leptin Receptor

Leptin and its receptor (LepR) are predominantly expressed in adipose tissue, with moderate levels found in the hypothalamus and endothelial cells. High-fat diets can significantly upregulate the LepR expression, potentially giving a false impression of an improved therapeutic delivery efficiency when targeting this receptor. To overcome these limitations, peptides derived from leptin, such as LP16, have been utilized for LepR targeting. These peptides help minimize competition with endogenous leptin, reducing the risk of side effects and improving delivery efficiency [234].

#### 5.4.6. Scavenger Receptor

Scavenger receptors (SRs) are widely expressed throughout the body, with their levels varying across different cell populations and tissues. A high expression is typically observed in macrophages as well as in organs such as the liver and heart, reflecting their roles in metabolic and immune processes. A unique characteristic of SRs is their lack of specificity for a single binding ligand. Instead, they recognize a diverse range of molecules, including LDL derivatives, proteoglycans, and residues from apoptotic cells. This versatility highlights their critical role in maintaining homeostasis and facilitating the clearance of cellular debris and foreign substances [234].

#### 5.4.7. Glutathione Transporters

Glutathione transporters (GSHTs) are predominantly situated on the luminal membrane of BBB endothelial cells, where they display a moderate to high expression. As glutathione (GSH) serves as a critical antioxidant within the body, utilizing it as a targeting ligand for drug delivery poses a minimal risk of side effects. This low toxicity, combined with the transporters’ strategic location and role, positions the GSHT as an effective and promising avenue for facilitating brain drug delivery via receptor-mediated transcytosis (RMT) [235].

The glutathione transporter stands out as a promising candidate for targeted drug delivery systems. A patented system involving glutathione-pegylated liposomes with doxorubicin has advanced to phase I/II clinical trials. This success is attributed to the well-established pharmacokinetics, tolerability in humans, and absence of significant limitations from the targeting ligand. This example could represent the first targeted delivery system to become clinically available, showcasing the potential of the glutathione transporter in medical applications [236,237].

#### 5.4.8. Diphtheria Toxin Receptor

The diphtheria toxin receptor (DtR) is expressed not only in BBB endothelial cells but also in glial and neuronal cells. It facilitates ligand internalization through a caveolae-dependent pathway, similar to the low-density lipoprotein receptor (LDLR). A unique feature of the DtR is that it is not targeted by any endogenous ligand, meaning that its targeting does not involve competition with natural ligands or the risk of disrupting the brain homeostasis. Exploiting the DtR for targeted drug delivery appears to be a promising strategy, and novel ligands for this receptor have been developed. CRM197, a non-toxic variant of the diphtheria toxin, has been used as a shuttle protein, demonstrating promising results in terms of a high efficacy in the CNS drug delivery and a low toxicity [238]. However, CRM197 is also utilized in certain vaccines to provide immunity against diphtheria, which could trigger the production of endogenous antibodies against the protein, potentially reducing its targeting efficiency [239].

#### 5.4.9. Efflux Pumps

Efflux pumps, part of the ATP-binding cassette (ABC) transporter family, are essential for exporting molecules from the brain. In animal models lacking these transporters, the BBB permeability increases significantly, compromising the barrier’s protective role. ABC transporters, such as P-glycoprotein (P-gp) and the breast cancer resistance protein (BCRP), are found throughout the body, with a high expression in tumor cells and brain endothelial cells. P-gp is involved in transporting a wide range of substrates, including chemotherapeutic agents, and is highly expressed at the BBB. The BCRP shares similar characteristics and is also expressed in brain capillaries and glioblastoma cells. These transporters present a challenge in drug delivery, as they can expel therapeutic agents before they reach their target. Strategies to overcome this include delivering the therapeutic alongside agents that silence ABC transporters, enhancing the drug accumulation at the BBB by inhibiting the efflux mechanisms [234].

### 5.5. Nanoparticles

Nanoparticles (NPs) have emerged as a promising alternative to enhance the pharmacokinetic properties of drugs, improving their solubility and ability to penetrate biological membranes. Given that CNS disorders such as AD and PD require lifelong treatments, strategies to achieve prolonged and sustained drug release are essential [240]. NPs range in optimal size for BBB permeability, ranging in size from 10 to 100 nm. Also, their surface charge influences the BBB crossing, with positively charged NPs showing better transcytosis but higher toxicity risks [241].

Surface modifications, such as the conjugation with ligands or polyethylene glycol (PEG), enhance the targeting and circulation time while reducing immune clearance. Functional ligands can enable receptor-mediated transcytosis, targeting specific receptors like transferrin or insulin receptors [242]. Inside the nanoparticles, complex shapes and surface modifications, including some like APO-E or PEG coatings, enhance their penetration. The nanoparticles are used in combination with other methods to improve the BBB permeability, such as CED or immunotherapy [243].

#### 5.5.1. Polymer-Based Nanoparticles

Polymer-based nanoparticles have emerged as drug delivery systems that stand out via their versatility and effectivity due to their stability, biocompatibility, biodegradability, ease of modifications of their active groups and manufacturing, high drug loading capacity, hydrophobic and hydrophilic drug transport capacity, non-immunogenic, low toxicity, and prolonged blood time circulation [242].

Polymeric NPs, like poly(lactic acid) and poly(butyl cyanoacrylate), can be easily modified with ligands such as Tf or PEG, improving the bioavailability and targeting of NPs to a specific brain tissue [244].

Inside the polymer-based nanoparticles, there are some delivery systems approved by the FDA, like donepezil (Aricept^®^), galantamine (Razadyne^®^), or rivastigmine (Exceleon^®^), which are all cholinesterase inhibitors to enhance the cognitive functioning of AD patients. Another FDA-approved drug is memantine (Namenda^®^), which is an antagonist of N-methyl-D-aspartate (NMDA) receptors and exhibits the capacity to reverse the cognitive decline of AD patients and reduces the behavioral symptoms and general functionality. Nevertheless, the efficacy of these drugs is limited due to their dose-dependent side effects, particularly at higher doses [245].

Dendrimers are hyper-branched polymers that represent a promising platform for delivering therapeutic agents to specific tissues or cells, minimizing off-target effects. Its potential arises from their ability to target specific sites; trigger drug release in response to stimuli such as the pH, temperature, or even enzymes; and penetrate the BBB. Like other polymeric nanoparticles, their multifunctional nature allows them to conjugate to different ligands, enhancing their properties and versatility. Dendrimers can be improved with acid-sensitive linkages to improve the controlled drug release in specific microenvironments [246].

Synthetic polymers like PANAM (polyamidoamine) dendrimers allow drug encapsulation in nanostructures smaller than 15 nm but face challenges like costs and toxicity. Natural polymer-based nanoparticles are explored as biodegradable alternatives [247].

#### 5.5.2. Lipid-Based Nanoparticles

Lipidic nanoparticles, such as liposomes, solid lipid nanoparticles, or even emulsions, have gained attention in AD due to their biocompatibility, versatility, and ability to encapsulate both hydrophobic and hydrophilic drugs. Moreover, these molecules can be modified to enhance the brain uptake or even prolong their stability or circulation time [248].

In a recent work by Han et al., peptide-functionalized lipid nanoparticles (pLNPs) designed for systemic delivery to the brain are explored to address the challenges posed by the BBB. In the study, four targeting peptides, RVG29, T7, AP2, and mApoE, were used to improve the brain-specific delivery. The results show that in vitro pLNPs enhanced the transfection efficiency in brain endothelial and neuronal cells. RVG29 and mApoE exhibited the highest improvements in cellular uptake. In vivo and ex vivo results show that pLNPs demonstrated significant brain-targeted mRNA delivery while reducing hepatic accumulation. RVG29 showed the highest neuronal transfection and minimal endothelial entrapment, suggesting its suitability for treating neurological disorders. The study highlights the need for tailored peptide designs to overcome barriers such as size constraints and the receptor downregulation in aging or diseases. LNPs or modified lipid nanoparticles have not only been demonstrated to cross the blood–brain barrier but also to target specific types of cells, and among them, we find the neurons. This work positions RVG29-functionalized pLNPs as a promising non-viral platform for neurological therapies [249].

Liposomes are easily synthesized lipid composites and are also easily modified. They are nanomaterials that can deliver compounds through different routes, such as oral, topical, parenteral, ocular, or even pulmonary routes. Liposomes have been widely explored as delivery systems for brain-targeted therapies. These spherical nanoparticles consist of an aqueous core surrounded by a phospholipid bilayer. While liposomes alone cannot cross the blood–brain barrier (BBB), they can increase drug concentrations in the brain by extending the circulation time. Additionally, liposomes are versatile, capable of encapsulating both hydrophilic and hydrophobic compounds. Conjugating liposomes with targeting ligands, such as peptides, can further enhance the drug efficacy. A study by dos Santos Rodrigues et al. demonstrated that liposomes conjugated with cell-penetrating peptides (CPPs) and transferrin (Tf) significantly improved the BBB penetration. In vivo experiments showed that TAT-Tf liposomes successfully delivered therapeutic DNA into the brains of mice. This CPP-functionalized liposome system shows great potential for targeted brain drug delivery.

Sealth liposomes are a second-generation type of liposomes designed to enhance their stability, prevent the opsonization by blood compounds, and reduce drug leakage. These liposomes typically use PEG on their surface to prolong the circulation time by avoiding premature removal through opsonization [250]. Advanced versions include functionalized liposomes, where specific targeting ligands, such as peptides, antibodies, or proteins, are added to enable active targeting to specific cells [251]. Additionally, there are smart liposomes that can be triggered by specific physical or chemical factors, like the pH, temperature, or light, to release their therapeutic cargo at the targeted site [252].

Niosomes are lipid-based nanoparticles made from non-ionic surfactants and cholesterol, offering a greater stability than liposomes. These are biocompatible and biodegradable nanomaterials that can deliver drugs through various routes, like oral, topical, or ocular routes, and can also be functionalized for targeted therapy. Niosomes are classified by their number of layers: multilamellar vesicles (MLVs), small unilamellar vesicles (SUVs), and large unilamellar vesicles (LUVs) [253].

Solid lipid nanoparticles (SLNs) are another lipid-based carrier system that is stable, biocompatible, and scalable, offering precise control over the drug size, loading, and release. SLNs protect drugs from degradation, improve bioavailability, and enable targeted delivery, reducing systemic side effects. These versatile carriers can encapsulate both hydrophilic and hydrophobic drugs, making them suitable for delivering various therapeutic agents, including anti-inflammatory drugs and genetic materials, which are crucial in personalized medicine [254].

Nanoemulsions have gained attention in recent years as a therapy for CNS diseases. They are fine dispersions of two immiscible liquids, typically oil-in-water (O/W) or water-in-oil (W/O), stabilized by surfactants. These systems contain very small droplets, ranging from 20 to 400 nm in size. Compared to traditional emulsions, nanoemulsions offer several advantages, including enhanced stability, a larger surface area, and faster absorption. Due to these properties, nanoemulsions are increasingly used in various drug delivery systems, including parenteral, oral, topical, and intranasal routes, making them a versatile option for efficient and targeted therapeutic applications [255,256].

Cubosomes are lipid-based nanoparticles with a unique cubic structure formed by the self-assembly of nonlamellar lipids. Their internal structure consists of a lipid bilayer and water nanochannels, making them biocompatible and capable of co-encapsulating both hydrophilic and hydrophobic compounds. They offer stability in biological environments and an ease of cellular uptake. Due to their cubic membrane structure, cubosomes provide enhanced drug encapsulation and controlled release, which is crucial for brain drug delivery. Research has shown that cubosomes can co-deliver therapeutic agents like curcumin and catalase to neuroblastoma cells, demonstrating neuroprotective effects. The efficiency of the cubosome uptake can vary depending on their surface properties and coatings, with different mechanisms observed for coated versus uncoated versions [248].

#### 5.5.3. Metallic Nanoparticles

Metal-based nanoparticles have a nanoscale size that allows them to penetrate biological membranes that are typically impermeable to macromolecules [257]. Functionalizing MNP surfaces with agents such as PEG enhances biocompatibility, extends circulation times by reducing the uptake by the mononuclear phagocyte system, and improves pharmacokinetic properties [258]. MNPs improve the solubility of hydrophobic drugs, prolong their bloodstream retention, and reduce rapid kidney excretion. When designed to release drugs in a controlled manner, MNPs can minimize the harm to normal cells, making them an ideal platform for precision medicine [259]. The magnetic properties of metallic nanoparticles (NPs) are widely utilized in innovative brain treatments because, by applying an external magnetic field, NPs can be precisely directed to target locations. Moreover, alternating magnetic fields generate heat within the NPs, inducing a hyperthermic effect called thermotherapy, which effectively destroys cancer cells in tissues like the brain [260].

Various MNP types, including gold, copper, silver, titanium, and palladium nanoparticles, are employed in targeted drug delivery. For instance, gold nanoparticles (AuNPs/GNPs) conjugated with the transactivator of transcription (TAT) peptide can effectively cross the blood–brain barrier (BBB). TAT-AuNPs can deliver therapeutic agents like doxorubicin (Dox) or imaging agents such as gadolinium (Gd^3+^). Studies reveal that GNPs disrupt tight junction (TJ) proteins, such as zonula occludens-1 and occludins, by inhibiting PKCζ phosphorylation, increasing the BBB permeability for drug delivery. Furthermore, GNPs can transport small interfering RNA (siRNA) molecules to cross the BBB and target oncoproteins [261,262,263,264].

#### 5.5.4. Quantum Dots

Quantum dots (QDs) are fluorescent nanocrystals used for imaging and drug delivery. They are semiconductors and composition-dependent fluorescent with tunable excitation and emission spectra, enabling multicolor and multitarget imaging. These properties allow QDs to visualize the brain vasculature, neurons, glial cells, and even individual receptors and ion channels. The conjugation with ligands such as transferrin (Tf) or TAT peptides enhances their ability to cross the blood–brain barrier (BBB) and target specific cells [265,266,267].

Carbon quantum dots (CQDs), known for their biocompatibility and photoluminescent properties, can cross the BBB using glucose transporters without additional targeting ligands. QDs can infiltrate the brain, enabling real-time tumor visualization for a preoperative diagnosis, intraoperative margin identification, and postoperative monitoring. For instance, QDs labeled with epidermal growth factor receptor (EGFR) antibodies selectively bind glioblastoma and oligodendroglioma tissues overexpressing the EGFR, allowing a single-cell-level visualization and precise tumor boundary demarcation. Conjugated QDs with therapeutic agents can combine imaging and therapy [268,269,270].

#### 5.5.5. Nanogels

Hydrogels are three-dimensional, hydrophilic polymeric structures capable of holding significant amounts of water without dissolving, mimicking biological tissues. Nanocomposite hydrogels incorporate nanoparticles (NPs) into their network, enabling them to combine hydrogel benefits, such as a fluid-like transport, low toxicity, serum stability, and uniformity with the advantages of NPs, including a small size, enhanced permeability, and suitability for intravenous administration [271,272].

Nanogels exhibit bioadhesiveness, biocompatibility, biodegradability, a high drug-loading capacity, and a responsiveness to specific stimuli like the pH, temperature, or ultrasound for controlled drug release. Moreover, they have shown promise in crossing the BBB. Thermosensitive nanogels and hydrogels are particularly noteworthy for neurological applications. These systems transition from a liquid to a gel at body temperature, enabling injectable formulations that conform to tissue shapes and provide localized, controlled drug release [273].

### 5.6. Antibodies

It is well-known that antibodies can reach the CNS under certain circumstances, but their clinical application requires optimization to overcome the inherent limitations of the BBB. Some advances in molecular engineering enable the design of antibodies with an enhanced BBB permeability. There are strategies, such as receptor-mediated transcytosis, that exploit endogenous transport mechanisms by targeting receptors like transferrin (TfR) and insulin receptors (IRs) on the endothelial surface [274].

Aducanumab was approved by the FDA in 2021, and its results show an amyloid removal linked to clinical benefits in AD. It is a human IgG1 antibody that has been shown to prevent aggregation and removes both the soluble and insoluble AB with cognitive benefits [275].

Lecanemab (Leqembi^®^) is a humanized IgG1 monoclonal antibody that has demonstrated the capacity to impede or retard the progression of AD. The antibody has been demonstrated to statistically reduce the amyloid markers in early AD, and results show that it moderately reduces the decline in measures of cognition and function, although it does not treat symptoms [26].

The temporary reaction of the immune system improves the paracellular permeability.

## 6. Developing Therapeutic Approaches

### 6.1. Epigenetics and Histone Modifications

The complex pathophysiological background of AD calls for the development of a multifaceted therapeutic approach [276,277]. Of the many mechanisms and therapeutic targets that are sought, epigenetic alterations have garnered much attention [278]. Altering gene expression patterns that influence important cellular processes involving inflammation, oxidative stress, and synaptic plasticity could be fruitful. DNA methylation and histone modifications as mediating interventions in AD that have the advantage of not requiring a change in the DNA sequence are seeing rapid progress [279]. Whether there are differences in the global DNA methylation in brain samples of populations with and without AD is controversial, with the literature reporting contradictory results [280,281]. However, there are changes in gene-specific DNA methylation in AD, such as the amyloid precursor protein (APP), β-secretase (BACE)1, MAPT, and apolipoprotein (APO)E genes [282].

Ruan et al. used high-throughput DNA methylation arrays on the peripheral blood of AD patients and healthy controls to find differentially methylated positions in those with AD and uncovered 18 hypermethylated positions (generally indicating a lower expression of the corresponding gene) and 23 hypomethylated positions (generally indicating a higher expression of the corresponding gene) [283]. They found that many of the abnormally methylated sites were located on genes related to the lipid metabolism and transport. Using their data, they constructed an AD risk prediction model.

Similarly, histone modifications such as methylation, acetylation, glycosylation, and ubiquitylation affect the access of transcription factors and other DNA-binding proteins to DNA. Histone acetylation changes in AD result in changes in synaptic plasticity and memory processing and storage [284].

Histone modifications of the ANK1 gene, which encodes ankyrin, a cell membrane protein that contributes to the cellular structure by linking the cell membrane to the spectrin cytoskeleton, have been investigated in AD. De Jager et al. linked the methylation state of ANK1 to its expression and connected ANK1 to AD susceptibility [285]. Lunnon et al. correlated the hypermethylation of ANK1 and AD neuropathologic changes in the entorhinal cortex in the postmortem human brain [286]. Smith et al. investigated the alteration of ANK1 histone modifications in AD [287]. They found a negative correlation between levels of the histone modification H3K4me3, affecting lysine residue 4 on histone 3, and DNA methylation in specific regions of the ANK1 gene. H3K4me3 is known to play a role in controlling gene transcription, and its association with ANK1 DNA methylation in AD brains further supports the role of epigenetic modifications in the AD pathology [288].

Histone acetyltransferases (HATs) add acetyl groups, and histone deacetylases (HDACs) remove the acetyl groups that were added. HDAC inhibition in murine models of AD can improve learning and memory [289,290,291,292,293]. The cytoplasmic enzyme HDAC6, which acts on non-histone proteins, has been of particular interest because of its effects on microtubules and because it has been found at elevated levels in the postmortem human hippocampus from AD patients [294,295]. A reduction in HDAC6 improves the cognitive function in AD mouse models [296]. It should be noted that HDAC6’s effects on tau and microtubules are an area of controversy, and some studies suggest that the prolonged loss of HDAC6 can cause increased tau acetylation, which would be a damaging effect [297,298].

In humans with AD, the PEGASUS phase 2a clinical trial compared a treatment combining the HDAC inhibitor sodium phenylbutyrate and the hydrophilic bile acid taurursodiol to a placebo over 24 weeks, focusing on the mechanistic targets and pathways. It found no cognitive improvements but reduced cerebrospinal fluid (CSF) levels of p-tau181 and total tau [299,300]. Additionally, the treatment resulted in lower levels of biomarkers of neuronal degeneration and gliosis. While there were no differences in clinical findings, possibly due to the small sample size and short treatment duration, biomarker results provide preliminary evidence that phenylbutyrate and taurursodiol engage AD pathways of neurodegeneration and should be further explored.

### 6.2. Mitophagy and Autophagy

Tre2/Bub2/Cdc16)-domain-15 (TBC1D15), a Rab GTPase, serves as an activating protein for RAB7A. RAB7A, in turn, plays a crucial role in lysosomal membrane repair and in controlling the trafficking from early endosomes to late endosomes to lysosomes. The mitochondrial morphology is disrupted in AD models, and TBC1D15 is typically highly expressed. The knockdown and silencing of TBC1D15 in microglia have been shown to promote lysosome function and autophagy, thus improving mitophagy [301,302]. By promoting autophagy, TBC1D15 knockdown may improve the amyloid-clearing capacity of microglia. Overall, controlling TBC1D15 may be an important strategy for improving the clearance of amyloid plaques and enhancing mitophagy, facilitating it as a therapeutic approach for AD.

### 6.3. Targeting ApoE

The pursuit of techniques to mitigate the effects of the APOE E4 allele, a major genetic risk determinant for late-onset AD, is gaining prominence, especially now that homozygosity is considered a direct cause [303]. Evidence suggests that APOE E4 contributes to the AD pathogenesis via multiple pathways involving neuroinflammation, synaptic dysfunction, neurofibrillary tangle formation, and amyloid-β accumulation [304]. Although no therapies directed at APOE are currently available to clinicians, several therapeutic approaches have been successful in mouse models expressing human APOE alleles, thereby increasing or decreasing APOE levels, blocking interactions between the APOE and amyloid-β peptide, and genetically switching APOE isoforms [305]. The use of immunotherapies to reduce APOE4 and alleviate amyloid-β peptide deposition is one possible avenue, and in vivo mouse experiments have shown benefits [306,307]. Gene therapies targeting APOE using the CRISPR/Cas9 genome-editing system are another therapeutic avenue that can be used to convert APOE4 to APOE3 or APOE2 genotypes [308,309].

The translation of the concept of changing the ApoE phenotype from preclinical to human clinical trials has been challenging, but one small human study has been performed. In clinical trial NCT03634007 (https://clinicaltrials.gov/study/NCT03634007 (accessed on 3 May 2025)), the DNA coding for neuroprotective ApoE2 was placed in an adenoviral vector and administered intrathecally into persons with mild cognitive impairment who were homozygous for ApoE4. Interim results showed the safety and tolerability of the treatment and a decrease in the CSF total tau and phosphorylated-tau181 in 9 of the 13 participants.

### 6.4. Stem Cells

Human cell-based models can be a great asset in testing treatments and gaining mechanistic insights [310]. Induced pluripotent stem cells (iPSCs) provide a cost-effective and time-efficient means of studying human neurons, freeing researchers from relying only on cell lines and animal models [311,312]. Differentiating human iPSCs originally derived from AD patients and healthy controls into cortical neurons provides a platform for evaluating new treatment options. There are several 2- and 3-dimensional stem cell models, such as 2-dimensional iPSC-derived neurons and 3-dimensional organoids [313,314,315,316]. An iPSC can be induced to differentiate into neurons and glial cells and then manipulated to recapitulate aspects of the AD pathophysiology [317].

Clinical trials based on exogenous stem cell therapy are in progress. The University of Texas Health Science Center, Houston, is beginning a Phase 1b/2a open-label study (NCT06775964) using autologous adipose-derived mesenchymal stem cells delivered by intravenous infusion to reduce neuroinflammation in AD [318]. Regeneration Biomedical has begun a similar trial (NCT05667649), in which Wnt-activated stem cells are administered directly into the lateral ventricles of the brain [319].

A preponderance of the therapeutic effects of stem cells is exerted through exosomes, small extracellular vesicles shed from cells [320]. Exosomes are composed of a lipid bilayer surrounding the cargo of the RNA, DNA, protein, and lipids reflecting their cell of origin. Exosomes can be engineered to carry specific content and are able to cross the blood–brain barrier and deliver therapeutics to the brain [321,322,323]. Animal studies are promising, but human testing will be critical [322,324].

Overall, despite the many proposed therapeutic strategies and clinical trials, there is no disease-modifying intervention that has yielded much success. Looking beyond amyloid and tau and expanding on the therapeutic avenues discussed is essential to creating breakthroughs [48].

## 7. Final Remarks

Biomarkers have emerged as powerful tools for enhancing the diagnostic accuracy of AD, offering clinicians the ability to detect the disease at its earliest stages and differentiate it from other causes of cognitive impairment.

Neuropsychological testing plays a vital role in the identification of biomarkers of AD by assessing cognitive performance in relation to neuroimaging, genetic, and biochemical measures. Memory and executive function tests have been shown to be valuable predictors of disease progression and can help identify individuals at a higher risk of developing AD.

The AD pathogenesis arises from a complex interplay of the amyloid and tau pathology, vascular dysfunction, neuroinflammation, oxidative stress, and lipid signaling dysregulation. Key molecular drivers such as BACE1, GSK3β, p38 MAPK, Cdk5, and PP2A orchestrate amyloidogenesis and tau hyperphosphorylation, while impaired autophagy and mitochondrial dysfunction exacerbate neuronal injury. These multifactorial mechanisms highlight the limitations of the traditional amyloid cascade hypothesis and underscore the need for integrated therapeutic approaches.

Emerging treatments include monoclonal antibodies (e.g., lecanemab and donanemab), BACE modulators, kinase inhibitors, and gene therapies targeting APOE. Developing drugs or delivery systems capable of crossing the BBB is essential for effectively treating AD. Without BBB-penetrant strategies, many promising therapeutics fail to reach therapeutic concentrations at their target sites. Therefore, innovative approaches such as nanoparticles, receptor-mediated transport, focused ultrasound, or intranasal delivery, as mentioned in this review, are increasingly being explored to bypass or transiently open the BBB. Advancing these technologies holds the potential to revolutionize AD therapy and enable truly disease-modifying interventions.

## Figures and Tables

**Table 1:** Key points of monoclonal antibodies in AD clinical trials. AD: Alzheimer’s Disease, ADAS-Cog: Alzheimer’s Disease Assessment Scale–Cognitive Subscale, ADCS-iADL: Alzheimer’s Disease Cooperative Study Activities of Daily Living Inventory Instrumental Subscale, CDR: Clinical Dementia Rating, CSF: Cerebrospinal Liquid, t: Total, MMSE: Mini Mental State Examination, N: Number, NA: Non-Available, NS: Nonsignificant, RBANS: Repeatable Battery for the Assessment of Neuropsychological Status, vs.: Versus, and w: Weeks.

Ref	Drug	Study Characteristics (Phase, Duration, n, Age Range)	Tools (Clinical Scales, Neuroimaging)	Biomarker Changes	Clinical/Neuropsychological Outcomes	Potential Relevance Both from Clinical and Biological Perspective
[23]	Donanemab (Amyloid-β)	Phase 3 76 w N = 1800 60–85	Gradual and progressive change in memory; tau PET and amyloid PET	Plasma pTau217: decreased (Log_10_−0.2) vs. placebo	iADRS: Better score compared to placebo	Donanemab significantly slowed clinical progression at 76 weeks in those with low/medium tau and in the combined low/medium and high tau pathology group according to PET biomarkers
[24]	Donanemab (Amyloid-β)	Phase 2 72 w N = 266 60–85	Gradual and progressive change in memory; positive amyloid and tau PET	Decreased plasma pTau217 (Log_10_−0.14) and GFAP: vs. placebo PlasmaAβ42/40, NFL: NS vs. placebo	iADRS: Better score vs. to placebo ADAS-Cog13: Inconclusive CDR-SB/ADCS-iADL/MMSE: NS vs. placebo	Plasma biomarkers pTau217 and glial fibrillary acidic protein levels were lower than in the placebo following donanemab, might provide additional evidence of early symptomatic AD pathology change through anti-amyloid therapy
[25]	Gantenerumab (Amyloid-β)	Phase 3 116 w N = 982 50–90	CSF tau/Aβ42, amyloid PET scan	Decreased CSF tTau, pTau181, Aβ40 vs. placebo CSF Aβ42: increased compared to placebo CSF NRGN: decreased vs. placebo CSF NFL: decreased vs. placebo Plasma pTau181: decreased vs. placebo Increased plasma Aβ42 vs. placebo CSF pTau181: −23.8% Plasma pTau181: −21%	CDR-SB: NS compared to placebo ADAS-Cog13: NS compared to placebo ADCS-ADL: NS compared to placebo	Gantenerumab led to a lower amyloid plaque burden than placebo at 116 weeks without clinical improvement
[26]	Lecanemab (Amyloid-β)	Phase 3 78 w N = 1766 50–90	Positive biomarker amyloid	Increased CSF Aβ42: vs. placebo Decreased CSF tTau and pTau181 vs. placebo Decreased CSF NRGN vs. placebo CSF Aβ40: NS vs. placebo CSF NFL: NS vs. placebo Increased plasma Aβ42/40 vs. placebo Decreased plasma pTau181, NFL, GFAP vs. placebo CSF pTau181: ~30 pg/mL compared to placebo −16 pg/mL compared to baseline Plasma pTau181: ~0.8 pg/mL	CDR-SB: Better score vs. placebo ADAS-Co14: Better score vs. placebo ADCOMS: Better score vs. placebo ADCS_MCI-ADL: Better score vs. placebo	Lecanemab reduced markers of amyloid in early AD and lowered cognitive decline
[27]	Efavirenz (ApoE, Lipids and Lipoprotein Receptors)	Phase 1 52 w N = 5 55–85	MMSE CDR	Increased plasma 24-OHC vs. baseline CSF Aβ40: NS compared to baseline CSF Aβ42: NS compared to baseline CSF tTau: NS compared to baseline CSF pTau181: NS compared to baseline	MoCA: NS compared to baseline	CYP46A1 activation by low-dose efavirenz increased brain cholesterol metabolism (as measured by high HC levels) in early AD
[28]	DNL747 (Anti-Inflammatory)	Phase 1 12 w N = 16 55–85	CSF Ab42 Amyloid PET	Decreased plasma PBMC pRIPK1 vs. placebo	No clinical endpoints included	RIPK1 in the CNS as a potential therapeutic tool for AD
[29]	Neflamapimod (Anti-Inflammatory)	Phase 2 24 w N = 161 55–85	CDR, MMSE; CSF Ab1–42, p-Tau, CT, MRI compatible with AD	Decreased CSF tTau, pTau181 vs. placebo CSF NRGN: NS compared to placebo CSF NFL: NS compared to placebo CSF Aβ40: NS compared to placebo CSF Aβ42: NS compared to placebo CSF pTau181: −2.1 pg/mL	HVLT-R/WMS immediate and delayed recall/CDR-SB/MMSE: NS compared to placebo	Neflamapimod treatment lowered CSF biomarkers of synaptic dysfunction but did not improve the cognitive scores
[30]	Gosuranemab(Anti-Tau)	Phase 2 238 w N = 654 50–80	Positive for amyloid beta	CSF Unbound N-terminal tau: Decreased in treatment compared to placebo CSF pTau181: Decreased in high dose treatment compared to placebo CSF tTau: Decreased in treatment compared to placebo CSF Aβ42: NS compared to placebo −7.1 pg/mL compared to baseline CSF pTau181: ~−25 pg/mL compared to placebo	CDR-SB/MMSE/ADCS-ADL/FAQ: NS compared to placebo group ADAS-Cog13: Significantly worse in treatment compared to placebo	No significant effects in cognitive and functional scores but reduced levels of CSF unbound N-terminal tau in gosuranemab group
[31]	Semorinemab(Anti-Tau)	Phase 2 72 w N = 273 50–85	MMSE CSF Ab42 amyloid PET	Increased plasma, tTau, pTau217 vs. placebo Decreased CSF tTau, pTau217, pTau181 vs. placebo CSF N-term Tau: NS compared to placebo Plasma pTau217: ~+88 pg/mL CSF pTau217: ~−50% CSF pTau181: ~−12%	ADAS-Cog11: Better score compared to placebo ADCS-ADL/CDR-SB/MMSE: NS compared to placebo	No treatment effects on functional scales nor on amyloid biomarkers
[32]	Tilavonemab (Anti-Tau)	Phase 2 96 w N = 453 55–85	MMSE CDR amyloid PET	NA	Worse score from baseline up to Week 96 in the Clinical Dementia Rating–Sum of Boxes (CDR-SB) score	No efficacy in treating patients with early AD
